# Moving research into practice: lessons from the US Agency for Healthcare Research and Quality's IDSRN program

**DOI:** 10.1186/1748-5908-2-9

**Published:** 2007-03-29

**Authors:** Marsha Gold, Erin Fries Taylor

**Affiliations:** 1 Mathematica Policy Research Inc., 600 Maryland Avenue SW, Suite 550, Washington, D.C., USA

## Abstract

**Background:**

The U.S. Agency for Healthcare Research and Quality's (AHRQ) Integrated Delivery Systems Research Network (IDSRN) program was established to foster public-private collaboration between health services researchers and health care delivery systems. Its broad goal was to link researchers and delivery systems to encourage implementation of research into practice. We evaluated the program to address two primary questions: 1) How successful was IDSRN in generating research findings that could be applied in practice? and 2) What factors facilitate or impede such success?

**Methods:**

We conducted in-person and telephone interviews with AHRQ staff and nine IDSRN partner organizations and their collaborators, reviewed program documents, analyzed projects funded through the program, and developed case studies of four IDSRN projects judged promising in supporting research implementation.

**Results:**

Participants reported that the IDSRN structure was valuable in creating closer ties between researchers and participating health systems. Of the 50 completed projects studied, 30 had an operational effect or use. Some kinds of projects were more successful than others in influencing operations. If certain conditions were met, a variety of partnership models successfully supported implementation. An internal champion was necessary for partnerships involving researchers based outside the delivery system. Case studies identified several factors important to success: responsiveness of project work to delivery system needs, ongoing funding to support multiple project phases, and development of applied products or tools that helped users see their operational relevance. Factors limiting success included limited project funding, competing demands on potential research users, and failure to reach the appropriate audience.

**Conclusion:**

Forging stronger partnerships between researchers and delivery systems has the potential to make research more relevant to users, but these benefits require clear goals and appropriate targeting of resources. Trade-offs are inevitable. The health services research community can best consider such trade-offs and set priorities if there is more dialogue to identify areas and approaches where such partnerships may have the most promise. Though it has unique features, the IDSRN experience is relevant to research implementation in diverse settings.

## Background

### Program context and rationale

Applied research aims to provide answers to "real world" questions. Whether that research is used in the real world and encourages innovation and change, however, has typically not been a major focus of attention in the research community. This situation is now beginning to change. In the United States, the Agency for Healthcare Research and Quality (AHRQ) – a major supporter of health services research – has redefined its mission to involve both the production *and use *of health services research "to improve the quality, safety, efficacy and effectiveness of health care for all Americans" [1]. In Canada, research organizations are studying how to transfer knowledge to decision makers [2] and are listening more to potential users of research in establishing priorities for health services research studies [3]. In the United Kingdom, the government is funding researchers to synthesize work across multiple disciplines to better support the use of that research in modernizing its National Health Service [4]. Such initiatives draw in different ways upon a variety of perspectives on how organizational change is promoted and integrated into health care [5-9].

An increasingly diverse array of programs exist to support interests in implementing research into practice as reflected in the cross-national initiatives referenced above. In the United States, programs like AHRQ's Translating Research into Practice (TRIP) have funded evaluations of diverse implementation strategies designed to implement clinical research findings into practice and identify strategies that are sustainable and reproducible [10]. Other programs, particularly recently, go beyond *researching *implementation to creation of structures to support ongoing partnerships between researchers and users of research in a variety of areas. Often the focus is on moving beyond specific systems to encourage more broad-based adoption that is scalable and supports demand-driven research that is responsive to user needs. Within AHRQ, examples of such programs include the Primary Care Based Research Network, Integrated Delivery Systems Research Network (IDSRN), Partnerships for Quality (PFQ), among others. Similarly, within the U.S. Department of Veterans Affairs, the Quality Enhancement Research Initiative (QUERI) has sought to implement research findings into improvements in patient care and systems. Such programs often aim to "shake up" current ways in which research is conceived and their form may be ambiguous – critical outcomes may be defined in vague terms and well-defined program logic models may not be articulated in an effort to provide flexibility for innovation. Such characteristics complicate traditional evaluation, yet some form of evaluation of such efforts remains essential to understanding what can be learned from current investments so that future efforts may be refined and more clearly articulated.

### Program goals and evaluation questions

This paper contributes to knowledge on the general topic of implementing research into practice by examining the experience of one initiative – AHRQ's Integrated Delivery Systems Research Network (IDSRN). As described in more detail later, IDSRN encourages formal partnerships between organized delivery systems and researchers to support work on operationally relevant studies to improve care delivery and systems. IDSRN's structure is based on the assumption that tying research to systems can result in research that is more relevant to user needs and more accessible to those users who reside outside the research community.

This paper attempts to answer two key research questions: 1) Was IDSRN successful in supporting the operational use of research findings and moving research into practice, either within IDSRN or externally? and 2) What characteristics or factors of teams or projects are associated with success (or lack of success) in moving research to practice? While aspects of IDSRN may be unique, the findings presented are broadly relevant to a research audience interested in the challenges of adapting research into practical applications.

In this paper, we first describe the IDSRN program broadly and the methods used to study it. (Additional details regarding the evaluation are provided by Gold et al [11].) Next we present a descriptive overview of the IDSRN teams and funded projects. We then discuss our findings, focusing first on the operational impact of IDSRN, as well as the factors that facilitated or impeded operational impact and implementation. Finally, we offer conclusions about IDSRN's strengths and weaknesses and draw broader implications from this work for those interested in moving research to practice.

### Program description

IDSRN was developed by AHRQ in 1999 to foster public-private collaboration in health services research and operations. The initial impetus of the program was to make data from private sector organizations involved in the financing and delivery of care more accessible to researchers by developing partnerships between researchers and those in operational delivery systems (e.g., health plans, medical systems). Shortly after IDSRN began, however, AHRQ's interests evolved and the agency sought to use IDSRN to develop ways of generating research findings and tools that would be applied in real world settings. Accordingly, IDSRN became a "learning laboratory" to conduct different types of projects, often identifying topics on an ad-hoc or opportunistic basis in response to emerging interests (within AHRQ or externally) or funding opportunities. This diversity and diffuse program definition is central to IDSRN.

In March 2000, AHRQ issued a request for proposals soliciting teams of partners and associated collaborators to participate in IDSRN. Teams were to marry research to practice by having researchers embedded in or collaborating with operational managed care plans, hospital-based integrated delivery systems, large multi-specialty groups, or safety net providers. In September 2000, AHRQ made awards to nine such consortia (see Table 1). Five of the nine were led by organizations with a direct connection to insurance or health services delivery systems, some with affiliated outside research partners. The other four teams were based outside of the delivery system in universities or research firms whose primary mission did not involve health care delivery, though they were affiliated with such entities. Teams selected for IDSRN were not awarded funding upon selection but did receive the (exclusive) right to respond to IDSRN requests for task orders – individual contracts awarded for specified projects.

**Table 1:** IDSRN partners and main collaborators

Led by operationally based partner
• The HMO Research Network, a longstanding network of research affiliates of large integrated and prepaid systems^a^
• Denver Health, a large integrated safety net provider system
• Weill Medical College/New York Presbyterian, a large urban medical system
• Marshfield Clinic, a rural group practice (with Project Hope)
• United Healthcare, a major national health insurer (through their Center for Health Care Policy and Evaluation and a subcontract with RAND)
Led by others
• Abt Associates (with Geisinger Health Systems)
• Emory University's Center for Health Outcomes and Quality (originally based at Aetna, with whom it continued to collaborate)
• Research Triangle International (RTI) (with multiple provider systems)
• University of Minnesota's Division of Health Services Research and Policy (with Blue Cross Blue Shield of Minnesota, the Medical Group Management Association and others)

^a ^See Vogt et al. [12] for more information on the HMO Research Network.

Most IDSRN projects were awarded on a competitive basis using a contract (rather than grant) mechanism. Under the IDSRN contract mechanism, applications were typically due a few weeks after AHRQ released a request for task order. Applications were then reviewed by AHRQ and moved through an expedited award process. This task order award process differs markedly in internal control and speed from the more traditional processes that AHRQ uses to award grants. Being selected for the IDSRN program meant that teams were eligible to compete to propose and carry out specific types of projects. AHRQ engaged in some dialogue with the teams to gather ideas for topics, although the process was not very structured. Projects also were solicited on topics that arose across AHRQ, or more broadly within the U.S. Department of Health and Human Services (HHS) (e.g., interest in bioterrorism or racial/ethnic disparities in health care).

During the period FY 2000–2003 (the period of our analysis), AHRQ awarded 58 separate IDSRN projects totaling $14.2 million, funded both through core AHRQ funds and through more dedicated sources, particularly in the areas of patient safety and bioterrorism. Projects were expected to produce relatively rapid results, with most contracts spanning 12 to 18 months.

IDSRN projects were diverse and spanned almost all of the areas of interest within AHRQ. Most awards were in five broad areas: quality improvement and patient safety; system capacity and emergency preparedness; cost, organization, and socioeconomics; health information technology; and data development. AHRQ solicited proposals for projects that typically had some operational link. Funding, timing, and AHRQ staff interest largely drove the composition of projects included in IDSRN.

## Methods

Our evaluation is descriptive in nature. It aims to help program sponsors and participants learn more about how the program and teams worked, with the goal of generating formative feedback that could be used to refine the program. Sponsors viewed such a design as appropriate given the limited knowledge of how to implement research into practice and the practical constraints on a more rigorous assessment. These included timing (the evaluation was not solicited until well after the program began), structure (the program was not designed to yield comparison groups or baseline data which could enhance assessment of impact), and funding (the evaluation was not funded at a level that supported primary data collection outside of interviews with IDSRN participants). These factors obviously constrain the scope and sophistication of the findings but are not surprising given the fact that IDSRN involved a broad-based and fluid initiative in an emerging area.

For this study, we examined the first four years of IDSRN over a 12-month period, starting in October 2003. We reviewed relevant documents, including AHRQ documents about the program overall and documents related to individual projects (e.g., proposals and final reports); analyzed characteristics of funded projects; and conducted semi-structured interviews with AHRQ staff (n = 26), as well as those involved in each of the nine funded IDSRN partner teams and their associated collaborators (n = 65).

We conducted the majority of interviews with AHRQ staff and partner/collaborator teams in-person, with the remainder conducted via telephone. Interview protocols for AHRQ staff focused on their role in IDSRN, the underlying rationale for the program, their perspectives on implementing research into practice, and their views of IDSRN's successes and challenges. The interviews with IDSRN teams included researchers and those with management responsibility within the associated delivery systems, the latter of whom were key intended audiences for the program. Protocols for IDSRN participants included questions on their perspectives on the program and rationale for participation, general experience with implementing research into practice, and experience with particular projects undertaken as part of IDSRN, including the factors that facilitated or impeded the operational impact of those projects.

Since IDSRN program resources were typically allocated on the basis of projects, we used this unit of analysis as a primary one for understanding the types of projects pursued and determining whether IDSRN led to changes in operations. (Sequentially-funded projects on the same topic were considered a single project.) Given IDSRN's evolving goals, we defined program success broadly as involving any operational impact, either within the organization in which the project work was conducted or through use of that work by other organizations. We categorized IDSRN projects in several ways to better understand the relationship between project characteristics and the likelihood that its findings would be used. For example, as discussed later, we identified subgroups of projects that employed a similar approach to implementation, at least implicitly (e.g., operationally-linked projects that assessed clinical interventions versus less research-oriented projects that developed web-based tools to improve care delivery). We also examined projects by the locus of any change or impact that occurred as a result (e.g., change in the project team's delivery system versus change in organizations outside of IDSRN).

Finally, to help identify what factors might have facilitated operational impact, we prepared four case studies highlighting projects that were identified as particularly successful in terms of operational impact based on interviews with IDSRN teams, AHRQ staff's perspectives on projects with the greatest impact, and available documents. We selected projects that reflected the diversity of work carried out under IDSRN, different collaborator/partner teams, and different funding sources. We then conducted additional interviews to gather information on exactly how and by whom the research or tool had been used.

### Overview of IDSRN teams and projects

#### IDSRN teams

Each of the nine IDSRN teams involved a lead organization and one or more collaborators that merged research skills with operational experience. In all but one case, the team was led by an entity whose mission was to conduct applied research. (The exception involved a team led by the CEO of a safety net system). Regardless of their base, these entities depended, at least in part, on "soft" money and, therefore, had more incentive than operational staff to promote IDSRN partnerships and to develop fundable proposals. Researchers based in operational systems either supplemented their own staff or not, depending on how they viewed the strength of their internal capacity, and the historical working relationships. Organizations' main reasons for participating were the opportunity to pursue applied research in operational settings and the perceived credibility and prestige of being part of IDSRN.

#### IDSRN projects

IDSRN projects were more expansive in their focus than more traditional health services research, with context and application being major concerns. However, projects also varied within IDSRN. Some IDSRN projects took more advantage of IDSRN's partnership between research and operations than others. Moreover, some projects relied strongly on an existing research base, while others were only loosely linked to the evidence base from the field. As shown in Table 2, about half of the IDSRN projects employed relatively traditional research methods that were applied to operational settings and needs. Within this category, we identified three somewhat diverse kinds of work:

**Table 2:** IDSRN awards FY 2000-FY 2003, by type

Type of project	Description	Total projects	Total funding	Examples	Potential link between research and practice	Challenges that influence value
**Research linked to operational settings**						

Research using IDS data	Take advantage of IDS administrative, claims, or other data to carry out applied health services research	12	$3,191,558	Racial differences in care outcomes; impact of payment policies on care in provider group with diverse characteristics; medication errors	Enhances the knowledge base for understanding how health systems work; gives access to data not otherwise available for research	Identifying questions for research that have potential for ultimate operational value; ability to generate findings that build on evidence base and are taking the "next step"
Operational data assessment and validation	Assess the capacity of systems to provide specific data, develop specific measures	4	$1,083,674	Capacity to conduct studies of race, ethnicity; operational validation of hospital quality measures; private sector data for national quality reporting.	Assesses one facet of infrastructure readiness to determine need for or make operational improvements	Uniqueness of individual systems; ability to move beyond assessment to make changes or take appropriate action
Clinical intervention and assessment	Patients in the IDS are involved in intervention; outcomes assessed	12	$2,769,120	Electronic order entry; otitis media practice guidelines; falls management tool	Identifies promising delivery interventions that work in practice	Evidence base for interventions; ability to generalize or bring to scale results

**Stretching traditional research boundaries**						

IDS systems analysis	Prospectively analyze IDS systems and flows to identify performance, needs, or potential areas for improvement	8	$1,958,126	Modeling link between care transitions and iatrogenic injury; assessing factors that influence diffusion of IT; assessing reasons for pneumonia hospitalization by Evercare patients	Uses delivery base to better understand problems or constraints and ways of intervening	Ability to generalize beyond a single system or point in time; follow-through on findings to identify and test improvements
Tool development	Develop web-based or other tools for care delivery or public health improvement	17	$3,957,230	Electronic order entry; otitis media practice guidelines; falls management tool	Identifies promising delivery interventions that work in practice	Evidence base for interventions; ability to generalize or bring to scale the results

**Other**						

Organizational studies using data outside of IDSRN	Projects that take advantage of IDSRN vehicle and participants to study issues relevant to IDS but not otherwise built on IDSRN unique qualities	3	$643,863	Quality provisions in MCO contracts; hospital-volume link; nursing home policies and quality	Addresses research questions that shed light on health care delivery organizations	Does not necessarily capitalize on IDSRN capacity
Dissemination infrastructure	Projects that aim to support infrastructure in various ways to encourage dissemination	2	$594,310	National network of medical group practices; leadership conference on patient safety	Improves channels of communication to get information out	Strategic importance of particular effort; relevance of infrastructure to other IDSRN work, AHRQ, or field

Source: Authors' classification based on awards information provided by AHRQ.

• *Operational data assessment and validation*: assessing the capacity of delivery systems to develop data and measures; this is one facet in organizational readiness to assess performance or identify improvements. An example is a study intended to validate AHRQ's quality indicators in specific operational settings.

• *Clinical intervention and assessment*: implementing clinical interventions based in the delivery system and evaluating their outcomes. An example is testing whether electronic order entry reduces medical errors.

• *Research using integrated delivery system (IDS) data*: conducting health services research within an operational organization that is other than evaluation of a clinical intervention. An example is using delivery system data to examine racial differences in health outcomes.

Most of the other projects stretched the boundaries of traditional research into what could be considered needs assessment or technical assistance, and may not necessarily be viewed as research. However, many projects in these categories explicitly focus on implementation in that they pushed towards operational change. This set of projects included work of two types:

• *IDS systems analysis*: assessing IDS operations to identify the need for improvement and appropriate areas for intervention. An example is a study of the reasons for hospitalizations for pneumonia among elderly patients (enrolled in an insurance product called Evercare) in order to identify how hospitalizations might be reduced.

• *Tool development*: creating new delivery or management improvement tools that provide a way for organizations to take action or make change in a specific way. A prominent example is the group of IDSRN projects focused on planning tools to aid in local responses to bioterrorism events.

A small number of remaining projects either involved research that did not seem to require or benefit by an affiliation with an operational delivery system (though it may have addressed issues of interest to those users), or projects that provided structure to support dissemination of findings without necessarily involving research. While the former category has little to no relationship with implementing research, the latter – while not research – does help promote adoption and knowledge transfer of research findings to additional settings.

## Results

### IDSRN's success as measured by effects on operations

Given IDSRN's broad goal of moving research to practice, our study's first research question focused on whether IDSRN was successful in promoting operational use of research findings (broadly defined). This is an important question since it relates to IDSRN's ability to use its design to generate work that was meaningful to users of research, in this case primarily organizations involved in delivering or financing health care. In this section, we first describe the perceived value of operational linkages among IDSRN participants and then provide an assessment of IDSRN's operational impact. The section concludes by discussing what we found about how participants in IDSRN viewed its goals since these are relevant to context and how success of IDSRN can or should be defined and measured.

### Value of operational linkages

Interviews with the executives in the IDSRN delivery organizations, as well as with researchers affiliated with each team, show support for the concept of linking researchers with potential users of research in team-based efforts. System executives said, for example, that when researchers are based in an operational system, this opens the door more readily to both formal and informal communications on project needs or implications. Researchers involved in IDSRN also said that they received personal benefits stemming from their ability to contribute to real-world questions and to learn more about operational systems. However, system executives also said that implementation of such linkages within IDSRN was not always as strong as it might be and that goals for immediate use of findings were naïve in light of constraints generated from both the research and operational worlds. For example, gaining the buy-in necessary to make operational changes within a health system may require substantial time and resources but once buy-in exists, leadership typically wants to move rapidly in implementation.

IDSRN also was valuable to AHRQ in tying it, as a major producer of research, to a group of potential users of research. AHRQ developed stronger ties with both researchers and executives within delivery systems that fall outside the university-based health services research community viewed as a core audience for investigator-initiated grants (the mainstay of AHRQ's research program). Through these ties, researchers gained access to private sector data for research. IDSRN also provided a base for AHRQ to collaborate with more operationally-based entities within HHS. Links with more operationally-based agencies have the potential to improve access to users of research who are outside of that research community, and to generate outside support for the research that operational agencies view as vital to their needs.

### Operational impact

The operational impact of IDSRN has been mixed, and widespread diffusion was rare over the period studied. Based on AHRQ's conceptualization of program impact, we identified and examined three types of operational impact among IDSRN projects:

• *Influenced actions within the IDSRN partner system*. This kind of change was operationally defined as a report (from either interviews or documents submitted to AHRQ) that the project had led to some operational change within the delivery system.

• *Influenced actions of other IDSRN partners*. This kind of change involved another of the core nine IDSRN partner teams being actively involved in an intervention or changed by it.

• *Influenced actions external to IDSRN*. This kind of change was defined as a report that the work had been used or considered by operational entities apart from the IDSRN partners/collaborators.

Table 3 describes short-term operational uses of project findings, including both local and more broadly based use of results. Among the 50 completed IDSRN projects, we found evidence that 30 had some operational effect or use as defined above. (Of the 58 projects awarded by the time of our evaluation, 50 had been completed so their short-term outcomes could be assessed.) Most often, operational use occurred within the system in which the research had been conducted. Findings from clinically based interventions positioned in systems were most likely to be used locally (within the delivery system), probably because of the immediate relevance of the findings. Both positive and negative results were of interest, as they illustrated what worked or did not work. In most cases, the findings in one operational system did not have more widespread use. There was little formal infrastructure in IDSRN to support more widespread dissemination, particularly outside of the nine teams participating in the program.

**Table 3:** IDSRN task order outcomes by project type, FY 2000- FY 2003

			Impact of task order on delivery system^a^				Other outcomes	
				
Type of project	Number of awards	Number complete	None^b^	Local	Other IDSRN teams	External	Peer-reviewed paper^c^	Follow-on task order awarded by AHRQ
**Total**	**58**	**50**	**20**	**19**	**1**	**10**	**12**	**9**

Tools	17^d^	15	3	4	1	6	1	4
Research with IDS data	12	11	8	2	0	1	5	1
Clinical intervention	12	9	2	7	0	1	1	0
IDS systems review	8	7	2	3	0	1	3	1
Data capacity	4	4	2	2	0	0	0	3
Research, no IDS data	3	3	3	0	0	0	2	0
Dissemination vehicle support	2	1	0	1	0	1	0	0

Source: MPR analysis of available information.

^a ^We classified impact based on evidence that the task order has had some operational impact (broadly defined) in the following settings: (1) locally within the delivery system in which the task order occurred, (2) among other delivery systems within IDSRN, or (3) external to IDSRN. In cases where a task order had an impact in multiple settings, we classified as highest setting (e.g., those with lcoal and external impacts were classified as external).

^b ^Reflects projects where there was no explicit evidence of impact. Because site visit time was limited, we could verify many but not all the outcomes for each task order with IDSRN partners/collaborators.

^c ^Number of tasks with 1+ publication. Only publications that are known to be published or accepted for pubilcation are included.

^d ^The 17 task orders reflect 12 separate bodies of work. The 17 include two sets of projects with an initial and follow-on task order and one set of four sequential projects.

Twenty of the 50 projects we assessed did not have identifiable operational uses. In some cases, such use perhaps was not a motivating factor for the study (e.g., studies that did not require systems data). But timeliness and the perception of limited generalizability also were barriers to the use of some study results. When studies were mounted in response to a particular problem, decision-makers often wanted to solve it rapidly and were unwilling to wait for research results. Because IDSRN used a task order vehicle (a form of government contract mechanism), the lag in mounting research was much shorter than under the traditional grant mechanism with external peer review – several months versus a year or more. However, this time frame still was not sufficient for many topics or user needs.

Some failure probably is inevitable for programs like IDSRN. Of the 20 studies that did not result in operational use, five led to peer-reviewed publications and one had findings that were judged of sufficient interest to warrant follow-up funding. Moreover, IDSRN teams found managerial interest in some findings even if they were not immediately relevant. For example, one project that presented findings on the influence of medical group structure, culture, and financial incentives on cost drew a standing-room-only audience at a meeting sponsored by the Medical Group Management Association (MGMA), which collaborated with one IDSRN partner.

### Other views of program goals and outcomes

For the purposes of this study, we gauged IDSRN success through evidence of operational impact because AHRQ wanted the program to be evaluated against such a goal. However, as discussed earlier, program goals evolved over time and were not clearly articulated at the start. Thus, it also is important to consider how participants in IDSRN perceived its goals as these bear on the interpretation of the findings on IDSRN outcomes.

Our interviews with AHRQ leaders at the outset of the evaluation showed that they tended to view program goals in broad terms that related to AHRQ's evolving view of its mission, without necessarily having a detailed or consistent sense of what this meant about how IDSRN and its associated projects were structured. Although AHRQ staff generally agreed that IDSRN should promote operational use and implementation of research, this goal was quite broadly defined. The agency funded a mix of projects whose ability to support operational change varied, particularly on a short-term basis. Moreover, AHRQ used IDSRN opportunistically, sponsoring bioterrorism and other projects when funding become available for such work. Such projects took advantage of available funding and provided IDSRN teams with a diverse array of possible projects, but did not necessarily yield a coherent set of initiatives designed to move research into practice. This meant that program decisions and structure were not necessarily strongly linked to the operational outcomes sought from the program.

AHRQ staff and IDSRN partners/collaborators also differed in their perceptions of what implementing research into practice means. Some interviewees (including both AHRQ staff and IDSRN partners/collaborators) viewed IDSRN as a "laboratory" that embeds research in real world settings, so that research is more sensitive to operational concerns and managers have better access to its results. Whether results are immediately relevant in a system was often of lesser concern than generating work that could ultimately benefit the health care system more generally. Others saw implementation differently, viewing IDSRN more as a vehicle "for pushing results out into the real world" and for testing applications on a more "rapid-cycle" basis than for conducting operationally relevant new research. For them, IDSRN was a program to complete cutting edge projects quickly and to get real input from real people in real time. Disagreements tended to be sharpest in evaluating the merits of highly user-driven research that might be applicable only in a single setting and supported by, at best, a limited body of available research. Senior executives in participating operational systems who were looking for relevant solutions might support this work. Yet some researchers based within systems perceived that, in their experience, there were risks in trying to conduct research that is too heavily focused on immediate utility in the system, as such applications were difficult to develop on a real-time basis and were more likely to yield results that may be proprietary, hard to share, or unique to a particular system.

### Factors that facilitate or impede moving research to practice

Our study's second research question focused on the factors that facilitate or impede operational use and moving research to practice in IDSRN. We examine first the effect of team organizational structure on operational use. Next, we present findings on the factors facilitating success, based on results from four case studies of diverse projects viewed as having strong outcomes. Finally, we describe what participants told us in interviews about the challenges and barriers they experienced that limited their ability to link research to operational needs and use.

### Effect of team structure

The IDSRN experience suggests that a variety of models of partnership may be feasible in integrating research into operational systems if certain conditions are met. However, the challenges associated with developing strong operational links vary across models. About half of the IDSRN teams were based on researchers that were embedded in the operational system. Not surprisingly, such partnerships were easier to form in organizations that already had such a pre-existing research entity and set of relationships. Existing internal research capacity within an operational system typically meant that the organization had already made a philosophical and financial commitment to such a linkage and had pre-existing channels of communication; therefore, as long as the structure remained stable, having that internal research capacity appeared to improve the chances for operational use and implementation.

Such partnerships were more challenging in teams where the research component was based outside the operational system. For such arrangements to work, outside researchers and systems needed to have or develop a strong working relationship. Having a prior history of working together helped make for more effective teams, partly because they were more fully aware of the capabilities of the partnering organization.

Internal champions also were key to the success of partnerships involving researchers based outside the operational system. Successful teams needed someone with sufficient senior standing in the operational system to generate commitments for collaboration and access to systems resources and data. An internal champion also brought necessary knowledge of internal corporate systems and operational characteristics and concerns, and the ability to interpret what these implied for the conduct of research. Hence, successful teams needed someone within the organization to help bridge the research and operational concerns and make projects happen.

Some teams involved outside researchers that worked with more than one operational system; such partnerships required more effort to coordinate. However, if the outside researchers invested the time to build strong relationships, this model seemed to have enhanced potential for generating scalable knowledge because data could be merged or interventions tested across systems. Such actions are relevant to operational leaders who spoke of concerns with "scalability" and "replicability." Unfortunately, however, the IDSRN structure did not allow the program to benefit fully from such multi-organization teams because projects were not funded at a level that supported work in multiple systems and because there were internal constraints to such collaborations, including incompatible data systems across organizations.

### Case study insights into factors facilitating success

To gain insight into what contributes to findings that are successfully implemented into practice, we looked in more depth at four projects for which there was some evidence of strong operational use or adoption. These included:

• *Bioterrorism tools*. Through a series of four task orders supported with bioterrorism funds from HHS, researchers at Weill Medical College of Cornell University developed two new interactive computer models to serve the needs of end-users in the public health and emergency response community: the Bioterrorism and Epidemic Outbreak Response Model (BERM), which estimates the minimum staff needed to operate a network of dispensing clinics in the event of an anthrax or smallpox epidemic, and the Regional Hospital Caseload Calculator, which calculates the rate of casualties produced by anthrax or plague releases based on a set of changeable assumptions. These tools have been adopted by many groups outside of IDSRN, including the federal government (e.g., the U.S. Centers for Disease Control and Prevention).

• *Improving culturally and linguistically appropriate services*. With support from the Centers for Medicare and Medicaid Services (CMS), IDSRN researchers affiliated with the Lovelace Clinic (part of the HMO Network [12]) in New Mexico developed guides to help managed care organizations plan quality improvement projects that are focused on enhancing culturally and linguistically appropriate services for enrollees in Medicare managed care. One guide focused on meeting the language needs of members with limited English proficiency, and the other on planning and assessment related to cultural competence. CMS sent copies of the guides to each Medicare plan and the guides also were disseminated via workgroups convened in multiple locations. In addition, they were used by others within and outside IDSRN. A follow-up project gathered information on the use of the guides.

• *Medication Information Transfer*. In a two-stage process, RTI worked with Providence Health System (Portland OR) to study how information on medications was transferred over the course of a hospital stay, identify six points of vulnerability, and model the reduction in medication errors that could be achieved using an e-medication list. In a second task order, the intervention was implemented by Providence and its effectiveness evaluated.

• *Racial and Ethnic Disparities in Quality*. Researchers at RAND worked with those in the Center for Health Care Policy and Evaluation at United HealthCare in a two-stage project that used claims and enrollment data from commercial and Medicare plans to investigate racial and ethnic differences in cardiovascular disease and diabetes. Under a second task order, the team developed a tool that health plans can use to graphically display and assess disparities. The tool also is being used to support the National Health Plan Collaborative to reduce racial and ethnic disparities, with funding from AHRQ and the Robert Wood Johnson Foundation.

We identified several common factors across these cases that appear to have played an important role in their operational success. First, each focused on a user need that was driven by internal and/or external requirements that meant there were important environmental and/or organizational reasons to make change. These reasons included concern over bioterrorism after September 11, 2001, Medicare's requirements for quality improvement projects related to cultural competence, pending requirements for hospital accreditation related to patient safety, or purchaser concerns with racial and ethnic disparities. Projects that focused on developing user-oriented tools for more broad-scale application were the most likely to be disseminated to broader audiences. The research base available to underpin these tools varied, and in some cases was relatively limited.

Second, each of the case study projects included some follow-on work – through additional IDSRN funding and other means – that was important to the implementation process. The follow-on work allowed project teams to take their inquiry to the next level and begin applying their research in more practical, operational ways, such as implementing an intervention or developing a tool.

Third, each of the four projects selected for case study addressed issues that had the potential to be of broad interest, a finding that related to the presence of environmental and organizational reasons for change. Fourth, in most cases, the project work included support for the development of fairly generic tools to help users apply them in other settings, which, as described above, increased the likelihood of dissemination.

### Factors that impede success

While the IDSRN structure had a number of characteristics that enhanced the communication and implementation of findings, participants reported significant barriers to the use and spread of research findings. Executives in operational agencies said they had only a limited amount of time to consider new innovation. Thus, the findings generated through IDSRN and similar work will compete with more immediate operational needs and priorities. For example, those at the operational level reported being overwhelmed with many externally imposed requirements of government, payers and others and constrained by limited funding and by information technology. Executives said there frequently are more ideas for potential adoption than resources to support them. Because local systems' buy-in was critical for use, executives favored findings that required only incremental change, and techniques developed outside the delivery system were sometimes suspect as not adaptable to the local context. Finally, some organizations were more receptive than others to the use of research and were more likely to have affiliated staff who championed its use.

There also are sizeable barriers to disseminating findings and promoting their use outside of the system in which they were generated. The IDSRN infrastructure assumed that IDSRN teams would be a natural audience for project findings, and its structure was developed to promote sharing within the network. However, IDSRN included diverse organizations that often did not view many of the others as important reference groups, with even seemingly similar organizations making distinctions among themselves (e.g. public versus university-based safety net providers). Because use of findings appears more likely when viewed as relevant in a particular setting, the advocacy of these findings by operational leaders who are respected by their peers is important in adoption. However IDSRN's structure provided little means to engage such individuals because its activity was led by researchers, whose target audience tends to be other researchers, regardless of their operational base. And because IDSRN funding was tied to projects, there was little flexibility to encourage other routes for dissemination.

The limited amount of funding for projects relative to the program's scope and objectives was the most universally cited limitation of IDSRN across all participants. One IDSRN participant aptly characterized IDSRN as having "champagne ideas on a beer budget." Many projects cost substantially more than the funds allocated by AHRQ and went forward only because the partners were willing or able to provide monetary or in-kind contributions in the form of information technology support, overtime work, or external financing of related overhead expenses. The willingness of systems to continue this support could change over time as environmental conditions or leadership change within organizations. Many said the long-run viability of this arrangement was problematic. On the other hand, despite participant concerns for the burden of in-kind and other support for IDSRN work, it is possible that delivery systems' own investment in the work may have been an important factor in promoting commitment and sustainability.

There also were program-wide barriers to widespread dissemination of project findings that might lead to broader uptake of results. Because almost all funding was allocated on a project-by-project basis, the structure of IDSRN provided a disincentive to fund a stream of work that might ultimately have an impact or to fund dissemination of work once projects were complete. Often completing one project was viewed as an opportunity for AHRQ to support a different area of need. In addition, AHRQ itself was limited in its ability to promote program goals because limited staff resources were available to plan such work and almost no resources were available to execute it.

## Discussion

### IDSRN's strengths and weaknesses

IDSRN clearly helped AHRQ move beyond its traditional focus on university-based health services research to encompass a broader set of researchers with more applied interests and affiliations – and to develop stronger links with operational organizations both outside and inside government. IDSRN also provided a vehicle for AHRQ to become more "nimble" in its funding and respond to emerging user needs that may stretch traditional research orientations. Given AHRQ's revised mission statement, these are important goals that have applicability far beyond the specifics of the IDSRN program.

Yet IDSRN also had weaknesses – organizational and conceptual – that detracted from its ability to move research to practice in concrete terms. Organizationally, there was too little infrastructure available within AHRQ, as well as the partner teams, to help identify priorities for work and support dissemination of findings. Conceptually, there also was too little time invested in thinking about how best to structure IDSRN work so that it was consistent with program goals. For example, a key strength of research involves its cumulative nature, with a diverse variety of studies reported over time. Synthesizing such studies has become an important way of generating evidence-based findings [13-15]. AHRQ could have better structured the IDSRN work to take advantage of this accumulated knowledge. Indeed some IDSRN participants perceived that projects were not always as closely linked to the evidence base in the field as was desirable. Moreover, sometimes project topics were only vaguely defined. While IDSRN allowed work to be responsive to systems and user needs, it did not necessarily result in projects that focused most heavily on areas where a solid research base existed and could be applied to support implementation, nor did it create a cohesive portfolio of work.

The impact of IDSRN also could have been enhanced by more emphasis on projects that lend themselves to spread in a variety of settings. Because scalability benefits from multiple tests, such projects are likely to cost more and, thus, AHRQ will be less able to support work in the wide variety of areas that the agency's audience advocates. Also, high-level executives on some teams who were attracted to IDSRN because of its ability to support important internal priorities may become less supportive of the program if they have a harder time gaining support for their projects. These kinds of trade-offs require consideration if the goal truly is to use limited funds to best support the implementation of research to practice.

### Implications for future efforts

IDSRN was managed as a series of mostly independent projects, with limited though increasing potential for follow-on work. But effective implementation arguably requires moving beyond single projects to develop longitudinal strategies that take maximum advantage of what health services research has to offer, while converting that knowledge into a form more accessible to users. Although work does not necessarily need to be sequenced in a linear fashion, or supported by the same sponsor, successful implementation requires the capacity to identify opportunities where research is relevant to practice, develop or identify findings from research that are relevant to those areas, generate tools and other vehicles for making findings relevant to practice, and work interactively with the practice community to make these tools both accessible and accepted by those in practice.

Taking into account the insights from this evaluation, AHRQ recently retooled IDSRN into a new version of the program – ACTION or Accelerating Change and Transformation of Organizations and Networks. ACTION builds heavily on the IDSRN model but refines it to build in the following: more infrastructure for user input to support demand-driven research at a program-wide level and within individual teams, an emphasis on projects that have broad applicability and potential scale, and the potential for drawing in external resources and sequencing task orders to allow sequenced work that is geared to priority areas [16]. As experience with ACTION grows, it may be possible to learn more about creating effective partnerships and focusing on collaborations that have the potential for the greatest yield.

### Broader implications for researchers interested in implementation

Researchers need to ponder the potential mismatch between real world problems, which tend to be complex and multi-dimensional, and the parsimony inherent in research, which may encourage simplification. The most important real world problems may benefit less from the insights of any single study or body of work than from the creative synthesis of that work across multiple bodies of work, disciplines, and approaches in ways that address practical questions rather than particular research questions. Regrettably, the development of such syntheses is still very limited, especially outside of the clinical arena, but there is growing interest in them [17,18].

Clearly there are important underlying tensions and issues in implementing research into practice that are difficult to address. These questions are as fundamental as: What is the mission of health services research? How should its success be measured? To what extent should implementability into practice be considered in identifying the priority of a given project or body of work? Is the measure of a study's worth the utility of its findings and if so, when and to whom? The findings from this study suggest that even those actively engaged in programs that seek such implementation have very diverse views about the answers to these questions.

Because available funds for health services research are tight throughout the world and the costs of implementing research into practice are not trivial, it could be useful to create a dialogue around these questions. We need to address not only the importance of implementing research but also what is being learned about alternative approaches to doing so on a broad scale, what may be reasonable to expect, and where the important priorities lie. To support this dialogue, it is vital that we learn as much as we can from existing experience with implementation in all its forms.
